# Hypercoagulability and dyslipidemia in membranous nephropathy with anti-phospholipase A2 receptor antibodies

**DOI:** 10.1080/0886022X.2024.2441392

**Published:** 2025-01-02

**Authors:** Michael Kitlinski, Zbigniew Heleniak, Alicja Dębska-Ślizień

**Affiliations:** Department of Nephrology, Transplantology and Internal Medicine, Medical University of Gdansk, Gdansk, Poland

Dear Editors, we eagerly read the study by Liu et al. which gives further insight on potential mechanisms for thrombogenesis in idiopathic membranous nephropathy (MN), by investigating the impact of anti-phospholipase A2 receptor (PLA2R) antibodies on hypercoagulability in MN [1]. The authors suggest that changes in the coagulation system might relate to anti-PLA2R antibodies. However, we believe crucial discussions in relation to the published literature on this matter to be left out. Moreover, weighing in the findings from Liu et al. our commentary further expands on how in MN with anti-PLA2R antibodies, cardiometabolic disturbances might play a larger role than hypercoagulability in the development of thromboembolic events (TEs).

As newer studies emerge, the biological mechanisms leading to TEs in MN seem to be more complex than initially thought. It has been consistently found that MN with anti-PLA2R antibodies (compared to without) increases the risk of developing TEs [2–4] – yet the exact reason is less elucidated. Hypercoagulability is a part of the multifactorial pathogenesis in MN-associated thrombogenesis, especially for TEs within the first months of MN onset. However, currently, available clinicopathological data does not support an apparent association between anti-PLA2R antibodies and a more severely impaired coagulation system. Liu et al. found MN subjects with anti-PLA2R antibodies to on average have higher fibrinogen levels, leading the authors to hypothesize that the presence of anti-PLA2R antibodies might exacerbate hypercoagulability [1]. On the other hand, another study published relatively simultaneously failed to reproduce these findings [3], as there was no significant difference in fibrinogen levels in MN patients with positive anti-PLA2R status as compared to those with negative anti-PLA2R status. Moreover, MN patients with anti-PLA2R antibodies that developed TEs had comparable levels of fibrinogen [3], which is also in accordance with a cohort including almost 400 MN patients with anti-PLA2R antibodies where neither platelet nor fibrinogen levels predicted the risk of venous TEs (VTEs) [2]. Yet there are still other biological mechanisms speculated to contribute to a more extensive hypercoagulable state in MN with anti-PLA2R antibodies (e.g. *via* intermediary pathways) [5], possibly not affecting conventional coagulation system tests to the same degree.

On the contrary, there is emerging evidence for prominent cardiometabolic dysfunction in the development of TEs among those with MN and anti-PLA2R antibodies. Also found in the above-mentioned paper by Liu et al. [1], MN with anti-PLA2R antibodies has been associated with higher total cholesterol (TC) and low-density lipoprotein (LDL) levels [2–4]. As thrombogenesis in the setting of MN seems to be influenced by anti-PLA2R antibodies, Henry et al. aimed to explore risk factors for VTEs in anti-PLA2R positive and anti-PLA2R negative subjects, respectively. Among subjects with MN and a positive anti-PLA2R status, they found for each 1 mmol/L increase in TC levels, an almost 50% increased likelihood of developing VTEs [4]. Despite the shortage of evidence-based data, treatment of hyperlipidemia is widely considered the norm in certain parts of the world when managing patients with nephrotic syndrome. It was demonstrated that statin therapy decreased the risk of developing VTEs in subjects with MN, even more so than anticoagulation measures [6]. Most of the light is shed on anticoagulation regimes when discussing preventive measures for TEs in MN. However, we believe that more focus should be targeted on dyslipidemia’s impact on TE-risk in patients with MN and anti-PLA2R antibodies, and if such patients would benefit from lipid-lowering therapy even if other risk factors for MN-associated thrombogenesis are absent.

We commend Liu et al. for providing further understanding of the impact of anti-PLA2R antibodies on the cardiometabolic and coagulative function in MN. Even if cardiometabolic disturbances might have a greater impact (than hypercoagulability) on thrombogenesis in the setting of MN and anti-PLA2R antibodies, both issues require further attention in future studies. Despite the discussions of anticoagulative prophylaxis in MN being the pivot in preventing TEs, we would like to as preliminary clinical measures remind and encourage physicians to routinely measure TC and LDL in all subjects with anti-PLA2R positive MN and initiate lipid-lowering therapies if necessary. Still, further studies on the interplay between anti-PLA2R antibodies, coagulation factors, and dyslipidemia will answer the question if MN subjects with positive anti-PLA2R status would benefit from more individualized anticoagulative and lipid-lowering therapies.
